# Slipped capital femoral epiphysis: a review of management in the hip impingement era

**DOI:** 10.1051/sicotj/2017018

**Published:** 2017-05-17

**Authors:** Mahmoud A. Mahran, Mostafa M. Baraka, Hany M. Hefny

**Affiliations:** 1 Division of Paediatric Orthopaedics and Limb Reconstruction Surgery, Department of Orthopaedic Surgery, Faculty of Medicine, Ain-Shams University 38 Abbasia Cairo 11566 Egypt

**Keywords:** SCFE, Slipped capital femoral epiphysis, Femoroacetabular impingement, Capital realignment, Osteonecrosis

## Abstract

Slipped capital femoral epiphysis (SCFE) remains the most common adolescent hip disorder. Most cases present with stable slips, and in situ fixation is the most commonly adopted treatment worldwide. The introduction of the concept of femoroacetabular impingement and subsequent studies have revealed SCFE-related hip impingement to be a significant pre-arthritic condition, and the previously suggested remodeling of the proximal femur after in situ fixation has been called into question. Complex proximal femoral osteotomies and more recently intra-articular procedures via surgical hip dislocation have been employed. The literature is still lacking a strong evidence to undertake such aggressive procedures. Moreover, the application of a particular procedure regarding the nature of the slip, being stable or unstable, the degree of the slip, and the condition of the physis has not been extensively described in the literature. The purpose of this article is to outline the SCFE-related hip impingement, to review the best evidence for the current treatment options for both stable and unstable slips, and to develop an algorithm for decision making.

## Introduction

Slipped capital femoral epiphysis (SCFE) remains the most common adolescent hip disorder [1–5]. Most cases present with stable slips [6], and in situ fixation using a single screw has been the traditional standard of care [7, 8]. Although associated with considerable safety and acceptable prognosis, recent literature highlights the outcomes of failure of in situ fixation to provide symptomatic relief on the clinical level and to stop the progressive hip osteoarthritis (OA) [5].

The remodeling potential of the proximal femur in SCFE is controversial. Advocates of in situ fixation believe that metaphyseal remodeling occurs with time, and that the residual deformity will not have clinically significant long-term sequelae [9–11]. On the other hand, many authors question this remodeling potential and recommend more complex surgeries [5, 7]. The recently introduced concept of femoroacetabular impingement (FAI) [12] and subsequent studies revealed considerable evidence of labral and articular cartilage damage in hips with untreated SCFE [5, 8], which ultimately lead to degenerative changes and OA. This made it mandatory to attempt to restore the proximal femoral anatomy.

Many surgical procedures have been advocated. Those comprised: restoration of the head-neck offset by osteochondroplasty (OCP), proximal femoral osteotomies (PFO), combined PFO and OCP. More recently, arthroscopic procedures and surgical hip dislocation (SHD) have been employed [13]. No current consensus exists to recommend a particular procedure regarding stable versus unstable slips, the degree of the slip, the timing of operation, and whether the physis is open or closed. All are items that have not been extensively discussed in the literature. The aim of this article is to review the best evidence for management of SCFE and its residual impingement, to discuss the various surgical options, and to develop an algorithm for decision making.

## SCFE: stable versus unstable

The most widely used classification for SCFE is based on physeal stability as proposed by Loder et al. [14]. A slip is considered stable if the child is able to walk on the affected limb, either with or without crutches. An unstable slip occurs when the child is not able to walk on the affected limb, even with crutches, and regardless of the duration of symptoms. This distinction is very important as the prognosis and treatment will vary considerably. Some authors have further elaborated the definition of unstable slips to include signs of radiographic instability. This has included a clear separation between the head and metaphysis, absence of metaphyseal remodeling, and incidental reduction of the slip angle by more than 10° during surgery [15].

## Osteonecrosis: the devastating complication

Although hip pain, limp, and impingement are regarded as poor functional outcomes, osteonecrosis (ON) is by far the most serious complication that could occur. In a study by Larson et al. [16], ON was found to be the most common reason for hip arthroplasty in patients with SCFE, in which moderately high revision rates were reported. Furthermore, long-term studies have shown that excellent function can be expected until the fifth decade if the hip can be stabilized without the occurrence of osteonecrosis [17].

Several risk factors have been investigated for ON, including physeal stability, slip angle, surgical intervention and fixation methods. The physeal stability was found to be the most important determinant for the occurrence of ON [24–26]. Loder et al. [14] reported a 47% incidence of osteonecrosis (14 of 30 patients) in unstable SCFE. In a series by Kennedy et al. [17], ON occurred in four hips with unstable SCFE (4/27 hips) and did not occur in hips with stable SCFE (0/272 hips). Kallio et al. [21] studied the effect of stability on epiphyseal vascularity. All stable slips had normal epiphyseal vascularity on bone scan, while unstable slips have shown avascularity. The potential mechanisms leading to ON include kinking of retinacular vessels, tamponade by the intracapsular hematoma or effusion, synovitis, timing of intervention and location of fixation devices within the epiphysis [18].

Various treatment modalities have been proposed to address these potential causes of ON in unstable slips. These include preoperative traction, gentle manipulative reduction, fixation methods, and timing of intervention (urgent intervention versus intentional delay) and open or percutaneous capsulotomy. None of these variables was found to significantly affect the rates of ON [17, 18].

## SCFE-related hip impingement

The earliest clinical description of SCFE was provided by Paré in 1572 [19, 20] without reference to the hip deformity in SCFE. Campbell’s first edition did not even mention the treatment of residual hip deformity in healed SCFE [22]. In 1936, Smith-Peterson described the “tilt deformity”. He postulated that primary hip OA that was previously considered idiopathic is caused by a subtle childhood disorder that passed unrecognized [23].

The remodeling potential of the proximal femur in SCFE is controversial in the literature. Wong-Chung and Strong [24] studied the amount of physeal remodeling after in situ pinning. Remodeling averaged 11.7° and it was greater in moderate and severe slips compared to mild slips. They recommended a period of at least two years between initial fixation and considering a realignment osteotomy. Conversely, Jones et al. [25] have found that the frequency of remodeling is inversely related to the slip severity. Although some authors have observed marked remodeling with significant improvement in pain and range of motion (ROM), the consensus observation is that the deformity will persist to adulthood [26].

Howorth postulated that SCFE is probably the most common cause of osteoarthritis of the hip [27]. Later on, Stulberg recognized the deformity secondary to SCFE and coined the term “pistol grip deformity” [28]. Ganz et al. [12] emphasized SCFE as a cause of FAI due to anterior metaphyseal impingement during walking and sitting. Abraham et al. [29] described a causal relationship between SCFE and OA, which was attributed to the prominent neck metaphysis and the malorientation of the femoral head articular cartilage.

The distorted anatomy results from the multiplanar slip, resulting in coxa vara and retroversion [30]. An extension deformity develops in which the femoral head displaces posteroinferiorly in relation to the femoral neck. The uncapped anterolateral neck metaphysis forms a bump, which impinges against the acetabular rim [12]. The retroversion manifests as decreased internal rotation, which together with the neck abutment may result in complete loss of internal rotation or more severely a fixed external rotation and out-toeing gait. The decreased hip flexion is due to a combination of metaphyseal abutment and extension deformity [32].

The degree of slippage has been correlated with the degree of impingement and loss of ROM [33, 34]. Hips with moderate slips experienced loss of ROM to the same extent as severe slips. Thus, the relative prominence of the neck metaphysis, rather than the degree of slip, was emphasized as being the major cause of impingement. Furthermore, in mild slips, the prominent metaphysis was found to have the major role in the development of FAI, while in moderate and severe slips, FAI was attributed primarily to head-neck deformity and secondarily to the prominent metaphysis. These data illustrate the importance of OCP to address the metaphyseal bump during surgery; in mild slips with an impinging metaphyseal bump, a simple OCP would be sufficient to relieve the impingement. While in moderate to severe slips, intertrochanteric osteotomy (ITO) has to be combined with OCP.

Acetabular morphology, notably retroversion, as a cause of FAI in SCFE was discussed in the literature. Controversies exist regarding being primary or secondary to SCFE. Sankar et al. [35] attributed the preexisting acetabular over-coverage to have a role in the etiology of SCFE. Retroversion was considered a predisposing factor to SCFE based on the detected retroversion of the contralateral normal hip [7]. On the contrary, Mamisch et al. [33] and Kordelle et al. [36] found no relation between the acetabular version with neither the degree of slippage nor range of motion (ROM) loss. They concluded that SCFE has no effect on acetabular development.

A direct causal relationship between FAI and OA has not yet been demonstrated and many asymptomatic hips in young males show radiologic findings of FAI [37]. Also, many studies concluded that hips with radiographic evidence of FAI might not develop OA on the long-term follow-up. Another confounding element is the fact that the efficacy of all of these current surgical procedures has been questioned [5]. This raises the question whether those hips require surgical intervention or skillful neglect.

Rab [38] proposed two different mechanical situations of impingement: impaction and inclusion. A very prominent metaphysis in a severe slip will cause “impaction” on the acetabular rim, causing limited range of motion, external rotation during gait, and difficulty sitting. The femoral head may be “levered” posteriorly out of the acetabulum, resulting in a pattern of posterior acetabular and labral injury. The second mechanical situation, “inclusion”, occurs in mild to moderate slips, where the metaphyseal prominence is not as severe or has decreased with remodeling. The rough and cartilage-free bony metaphysis injures the acetabular cartilage with routine motion. The inclusion theory defeats the previous impression that metaphyseal remodeling is an advantage to range of motion and function.

In a review by Sink et al. [8], surgical hip dislocation (SHD) was performed in 39 hips (eight mild, 20 moderate, and 11 severe) for chronic SCFE with impingement symptoms. Labral and articular cartilage injuries were present in 34 and 33 hips respectively. In agreement with the Rab model, the location of labral and articular cartilage injuries was consistent with the impingement by the anterolateral metaphyseal bump. Similarly in a series by Leunig et al. [39], labral and cartilage damage was present in 14 consecutive hips. They described a “semilunar” cartilage injury ranging from chondromalacia, cleavage to full-thickness defects.

Castan et al. [5] followed 121 patients with stable slips that were fixed in situ for a minimum of 20 years. Ninety-six patients had clinical and radiographic signs of FAI and all patients developed radiographic signs of OA. The authors noted that the degree of OA was directly related to the initial slip angle and that even mild slips had resulted in FAI. The most significant predictor of OA was found to be the alpha angle of Stoecklin et al. [40]. In a similar series by Dodds et al. [31], the alpha angle was the most reliable predictor of FAI, but they did not find a clinically significant correlation between the initial slip angle and the development of symptomatic impingement.

## Current treatment options

Once diagnosed, the treatment of SCFE aims at stopping slip progression, relieving impingement, and preventing or delaying OA. Equally important is to avoid complications, notably ON, chondrolysis, slip progression, and fixation problems [13, 41], many of which could be surgeon-related. Different treatment modalities evolved. The range of treatment comprised methods to stabilize the epiphysis, with or without epiphysiodesis [42, 43], methods to reduce the slip, and methods to relieve the impingement.

A systematic review by Loder and Dietz [13] was performed in 2012. The levels of evidence for nearly all these studies are levels III and IV. This evidence indicated that the best method of treatment for the stable SCFE is in situ pinning with a single central screw. This procedure was associated with the lowest incidence of complications. Up to date, there is no evidence to support a proven clinical benefit of osteotomy, arthroscopy, or SHD over in situ fixation for stable slips. Long-term prospective data are required to decide the efficacy of the more elaborate procedures over in situ fixation.

In unstable SCFE, the strength of the existing literature is limited. This could be attributed to many factors. First, the definition of stability, although clearly displayed by Loder, has been subjected to various interpretations. Second, the temporal interval during which ON is investigated was not found to be consistent in many studies. In the original study by Loder et al., a rate of 33% ON after one year has jumped to 47% after two years. Hence, a minimum follow-up period of two years was recommended. Third, the literature concerning the treatment and results of unstable SCFE is retrospective level IV data [18] lacking a standardized validated outcome [13]. To add to the complexity, more confounding variables were found in unstable slips. Aspects like the timing to intervention, the use of a single over multiple screws for fixation, capsular decompression, and the methods of reduction [13] were employed. These data explain why the best evidence for treating unstable slips is still not yet known.

Treatment modalities to relieve FAI can be grouped into proximal femoral osteotomies (PFO), OCP, and a combination of both PFO and OCP. In addition, the roles of surgical hip dislocation and hip arthroscopy are discussed.

## Proximal femoral osteotomies

A PFO realigns the proximal femur, moving away the prominent neck metaphysis to increase the ROM before impingement occurs and reorients the good quality articular cartilage of the central femoral head back into the acetabular dome [7, 29]. Three PFO levels have been identified: subcapital, basal neck, and intertrochanteric. The more proximal the osteotomy the greater the degree of correction, but the higher rates of ON due to the vicinity to the femoral head blood supply [41].

The timing of PFO in relation to stabilization of the epiphysis has been controversial in the literature. Hosalkar et al. [7] recommended a period of 6–12 months after in situ pinning for stable slips, as a more conservative approach with fewer potential risks. Witbreuk et al. [41] recommended one-stage osteotomy plus epiphysiodesis in the management of moderate and severe slips, as this would relieve the impingement at an early stage and avoid the subsequent acetabular damage. Other studies [24, 44] advocated the remodeling potential and recommended initial epiphysiodesis followed by corrective osteotomy after physeal closure or when symptomatic impingement occurs.

### Intertrochanteric osteotomy (ITO)

Southwick [45] originally described a biplane osteotomy at the level of the lesser trochanter, based on measurements of the epiphyseal-shaft angles. Via an anterolateral approach, Southwick performed an anterolateral-based wedge creating a compensatory valgus-flexion deformity in 55 hips with SCFE. He reported good functional results with no ON. In 1966, Imhauser described a triplane intertrochanteric osteotomy, slightly proximal to the Southwick osteotomy [41], realigning the head to shaft by creating valgus, flexion and derotation. Many studies compared subcapital and intertrochanteric osteotomies and concluded that intertrochanteric osteotomies are safe, effective, and reproducible realignment procedures [41, 46].

A number of clinical trials [41, 44, 47–50] evaluated the outcome after ITO, many of which have shown good functional outcome regarding pain, ROM, and the onset of radiographic OA. The use of different hip scoring systems and different methods of radiographic evaluation makes it difficult to compare these studies. It is noted that that the percentage of patients with good to excellent outcome is inversely proportional to the duration of follow-up, with better results in the early years of follow-up. Diab et al. [44] compared the functional outcome of ITO and in situ pinning. Although the ITO improved the ROM, they found no significant differences in the short-term outcome in both groups.

Many centers adopted the ITO; their published data has shown good results regarding the ON rates. Salvati et al. [51] performed 24 Southwick osteotomies in patients with chronic severe slips, after a follow-up range of 2–10 years, one patient had ON. In a prospective series by Coppola et al. [52], the procedure was performed on 22 hips and they reported no cases of ON after an average follow-up of 22 years; 36.4% showed radiographic evidence of OA, but were asymptomatic. Chondrolysis was a recorded complication in a number of studies [52–54], many authors attribute this to postoperative immobilization in plaster and recommend early ROM after the procedure. ITOs create a reverse deformity in the head-shaft area, which may complicate the insertion of a femoral stem, in case a total hip arthroplasty is later undertaken. However, a number of studies reported good remodeling of the proximal femur after blade plate removal [52].

### Basal neck osteotomy

The earliest description was provided by Barmada in 1964 [55]. Later in 1976, Kramer et al. [56] described a “compensating osteotomy” at the level of the base of the femoral neck to correct varus and retroversion in moderate to severe SCFE. They highlighted the benefits of this osteotomy as being extracapsular, distal to the main blood supply of the femoral head, and hence less risk of ON. Also, compared to the more distal intertrochanteric or subtrochanteric osteotomies, it restores the mechanical relationship of the greater trochanter and the abductor muscles instead of introducing a second deformity. It was emphasized that a short lever arm created by the coxa vara is less important than the posterior displacement of the greater trochanter created by the retroversion. Accordingly, an intertrochanteric or a subtrochanteric osteotomy, which realigns the deformity distal to the greater trochanter, would have a less compensating effect on the abductor muscles compared to a basal neck osteotomy performed just above the greater trochanter.

Nonetheless, we did not find so many reports of this osteotomy in the literature. In the series of 55 patients by Kramer et al., 48 cases were reported to have good to excellent outcome, with only nine cases reporting poor outcome because of ON or chondrolysis. Abraham et al. [55] performed basal neck osteotomy on 36 hips and reported 90% good to excellent outcome and no cases of ON. In spite of encouraging clinical results and minimal risk of ON, limited correction could be achieved. Barmada et al. [57] identified the maximum correction to be 55° of retroversion and 50° of varus deformity. Thus, residual impingement might remain when correcting severe slips. On the contrary, the retrospective series by El-Mowafi et al. [58] found no significant difference comparing basal neck and Southwick osteotomies, at an average 3.5 years follow-up. They considered both procedures to be equally safe and effective.

### Subcapital osteotomy

Subcapital osteotomies appear to be the only method capable of restoring a normal or a near normal hip anatomy. It offers the greatest correction since it is performed at the level of the slip, but at the expense of high risk of ON due to its proximity to the postero-superior retinacular vessels.

The technique of subcapital osteotomy was originally proposed by Dunn [59] in 1964. He emphasized the importance of reducing the head onto the neck without tension on the retinacular vessels. Through a lateral approach with a trochanteric osteotomy to provide a clear field of vision, he described two maneuvers. First in acute on top of chronic slips, where the epiphysis is loosened from the neck, he advised neck shortening by a trapezoid osteotomy. Second, in moderate and severe slips with open physis, he described a technique of gently mobilizing the epiphysis through the physis then reflecting the retinaculum away from the posterior aspect of the neck, followed by resecting the posterior callus and trimming the neck stump. The fact that epiphyseal mobilization with reasonable ease and safety needs an open physis was highlighted. Dunn did not attempt neck osteotomy in any case with a closed physis, as it would endanger the blood supply to the femoral head. Out of the 23 patients who underwent subcapital osteotomy by Dunn, only one case developed ON [59]. Subsequent studies adopted Dunn procedure with ON rates ranging from 12 to 17% [60].

## Femoral head-neck osteochondroplasty

OCP can be performed by an anterolateral, anterior, mini-access anterior approach, or by surgical dislocation. More recently, arthroscopic procedures have been employed. Heyman et al. [61] provided the earliest description of OCP in SCFE, performed simultaneously with in situ pinning, through an anterolateral open approach. They referred to this procedure as a conservative operative treatment. The original work by Dunn has recommended an ITO with excising the neck bump in cases with moderate to severe slips and closed physis [59]. In moderate to severe slips, OCP alone would not only lead to increased hip range of motion, but would also allow for the thinner peripheral femoral head articular cartilage and prominent metaphysis to articulate even more extensively with the acetabulum [55]. Again this supports the role of the deformity, in addition to the bump in the pathogenesis of impingement.

The work by Mamisch et al. [33] highlighted that in moderate to severe slips, an ITO may improve the ROM, but those patients may need an additional OCP to relieve the whole impingement process. Bali et al. [62] compared the results of two groups of patients who underwent ITO with and without OCP. The marked clinical improvement in former group was found to be statistically significant. Moreover, this combination was found to be safe regarding the occurrence of ON and chondrolysis.

## The role of surgical hip dislocation

Ganz and coworkers [63] have described an approach to dislocate the hip safely with no risk of ON. Through a trochanteric flip osteotomy, the hip is dislocated anteriorly, and an extended retinacular flap is carefully developed allowing safe mobilization of the epiphysis, the so-called “modified Dunn procedure” with adequate inspection of intraarticular pathology and dynamic assessment of femoroacetabular contact. SHD approach can be utilized in SCFE in many aspects:

### (a) SHD for modified Dunn procedure (Table 1)

The main advantage of this procedure is the prevention of impingement through anatomic or near anatomic restoration of the proximal femoral anatomy [64]. The technique as described by Leunig et al. [65] entails performing an extended retinacular flap to expose the posterior aspect of the femoral neck followed by resecting the posterior callus. This is followed by trimming of the femoral neck and physeal cartilage to reduce the head onto the neck without any tension on the posterior retinacular flap. Many slips diagnosed as unstable on clinical basis have been shown to demonstrate extensive posterior callus formation [66]. This has been linked to decreased epiphyseal blood supply. Resection of this callus was emphasized to restore epiphyseal perfusion [67]. The presence of this callus prohibits even gentle closed reduction by many authors [59, 67].

**Table 1.** Summary of the results of studies undertaken SHD (modified Dunn procedure).

Authors	Year	Patient No.	Mean age	Stable vs. unstable	Physis (open /closed)	Mean (lat.) slip angle pre	Mean slip angle post	Mean follow up (months)	ON rate number (%)	Other complications (number)
Leunig et al. [65]	2007	30	13 years	Stable (24)	N/A	50	N/A	55	No patients (0)	HO *(1), screw failure (2), K-wire failure (1)
				Unstable (6)						
Ziebarth et al. [68]	2009	40	12.8 years	Stable (28)	N/A	56.6	8.6°	45.6	No patients (0)	HO (3), residual FAI (1), delayed union (3), wire breakage (3)
				Unstable (12)						
Slongo et al. [73]	2010	23	11.9 years	Stable (6)	N/A	47.6°	4.6°	29	2 Patients (8.6)	Severe OA (1), prominent K-wire (1)
				Unstable (17)						
Sucato and Podeszwa [74]	2010	15	12.5 years	All unstable (15)	–	N/A	N/A	–	1 Patient (6.7)	Fixation failure (2)
Alves et al. [70]	2012	6	12.5 years	All unstable (6)	–	37.0°	N/A	20.4	4 Patients (66.7)	Fixation failure (2)
Madan et al. [71]	2013	28	12.9 years	Stable (11)	Open (24)	59°	7.5°	38.6	2 Patients (7.14)	Slip progression (1)
				Unstable (17)	Closed (4)					
Sankar et al. [69]	2013	27		All unstable (27)	–	N/A	6°	22.3	7 Patients (26)	Fixation failure (4)
Souder et al. [66]	2014	17	12.2 years	Stable (10) Unstable (7)	N/A	N/A	N/A	15.6	4 Patients (23.5)	Chondrolysis (1)
Mohamadean et al. [75]	2014	33	13.8 years	N/A	N/A	43°	4.2°	24.2	2 patients (6)	Deep infection (1)
Upasani et al. [64]	2014	43	12.0 years	Stable (17)	N/A	N/A	N/A	21.2	10 Patients (23)	Femoral neck nonunion (4), postoperative dislocation (2), HO (2), fixation failure (2), stitch abscess (1)
				Unstable (26)						
Bali et al. [76]	2014	8	17.8 years	Stable (8)	Closed (8)	36.6°	15.4°	41	No patients (0)	Femoral neck nonunion (2)

* HO (heterotopic ossification).

Having all the complications aside, the ON is of particular concern (Table 1). The earliest two reports of this procedure [65, 68] have demonstrated no cases of ON, this has been attributed to careful visualization and protection of the retinacular vessels during reduction of the capital epiphysis. Although this should theoretically reduce the ON rates, subsequent studies have reported ON rates up to 26% [69]. In one study [64], the surgeon’s experience with the procedure was found to be a statistically significant factor in the incidence of complications. Many studies reported this procedure to be technically demanding with a long learning curve, besides the complications related to the trochanteric osteotomy, heterotrophic ossification, fixation failures, slip progression, postoperative dislocation, and sciatic nerve palsy were reported [70, 71].

The evidence for the best indication for the modified Dunn procedure is not yet known. Concerning physeal stability, all studies describing the modified Dunn procedure reported higher incidence of ON among the unstable group compared to the stable group [64, 66, 71]. The procedure was reported to be relatively easier when performed for unstable slips compared to stable slips [72]. The physeal separation allows easier mobilization of the femoral head, less trimming of the femoral neck.

Femoral neck shortening was reported to account for subtle instability of the hip joint, thereby converting a hip pathology from impingement into instability [13]. In the authors’ experience with unstable slips, resection of the posterior callus after SHD and a retinacular flap was found to reduce the tension on the retinaculum enough to avoid neck shortening in most cases.

Concerning physeal closure, few studies, in agreement with the original Dunn manuscript reported technical difficulty and increased ON rates with epiphyseal manipulation in healed slips with a closed or a partially closed physes [72]. The presence of physeal cleavage facilitates subcapital realignment. These studies recommended an ITO in slips with closed physis [59, 67].

### (b) SHD for OCP or combined ITO and OCP

Utilizing the SHD approach for combined ITO and OCP is infrequently discussed in the literature. In a series by Rebello et al. [72], 23 patients underwent SHD and ITO, of which 15 underwent combined ITO and OCP, and eight underwent ITO alone. They reported that combined ITO and OCP could decrease the amount of correction needed from the ITO thus minimizing the resultant proximal femoral deformity and facilitating future total hip replacement.

Spencer et al. [77] evaluated the outcomes of 19 patients with healed slips who underwent SHD for either OCP alone or combined OCP and ITO. After an average follow-up of 12 months, no cases developed ON and clinical improvement was reported to be higher among patients in the combined group. Patients with chondral flaps were reported to have less improvement. The incidence of complications of SHD approach combined with an osteotomy is substantially high. Added to this reported technical difficulty, most studies recommend these procedures to be performed in dedicated centers with sufficient expertise in the field of open hip preservation surgery [13, 72].

## The role of hip arthroscopy

Futami et al. [78] in 1992 performed hip arthroscopy on five patients with SCFE prior to in situ fixation. They recommended performing arthroscopy together with in situ fixation to relieve pain and allow early exercise in patients with slips.

Many authors adopted the arthroscopic treatment of FAI in cases with mild to moderate slips (Table 2). The arthroscopic approaches provide a less extensive, minimally invasive approach to the removal of impinging structures in mild slips [34]. The fact that the most pronounced deformity is found at the antero-superior and superior level, makes OCP easily accessible by arthroscopy using the standard portals [79–81].

**Table 2.** Summary of the results of arthroscopic osteochondroplasty.

Authors	Year	Patients No.	Arthroscopy and in situ fixation	Mean slip angle	Follow up (months)	Mean α-angle			Mean mHHS	
						Pre	Post	Correction	Pre	Post
Leunig et al. [82]	2010	3	Single session	19.6°	14.5	86.0°	48.6°	37.3°	N/A	N/A
Chen et al. [83]	2014	34	N/A	Mild to moderate	22	88.2°	56.9°	31.3°	N/A	N/A
Wylie et al. [84]	2015	9	58.6 Months after fixation	Mild to moderate	28.6	75°	46°	29°	63.6	91.4
Tscholl et al. [79]	2016	14	N/A	16°	16	57°	37°	20°	N/A	N/A
Basheer et al. [81]	2016	18	Arthroscopy after fixation	Mild to moderate	29	–	–	–	56.2	75.1

Clohisy et al. [85] presented a technique for management of cam lesions which combines mini-open anterior approach and arthroscopy with good short-term results. Their technique was found to overcome the disadvantages with performing arthroscopy alone; inadequate osseous debridement and the risk of bony debris becoming entrapped in the joint, and inadequate osseous debridement.

The role of hip arthroscopy is still limited. In moderate to severe slips, although restoring a normal alpha angle seems possible in moderate slips, the risk of femoral neck thinning and fracture remains a problem [34]. The small number of studies makes it difficult to recommend with or against arthroscopy. This makes modified Dunn procedure or ITO more favorable approaches in moderate (and severe) slips [79].

Besides cam, hip arthroscopy cannot address all elements of FAI namely, acetabular retroversion, femoral retroversion, and coxa profunda [35]. Moreover, morbid obesity, scarring from previous surgery, and the presence of screws in the anterior neck presented challenges to the arthroscopic technique [83]. As with open OCP, the timing of arthroscopic OCP in relation to in situ fixation is still controversial. Whether they should be performed as a single stage or a two-staged procedure is still not clarified in the literature [79, 82, 86].

## The authors’ proposed algorithm

**Figure 1.**
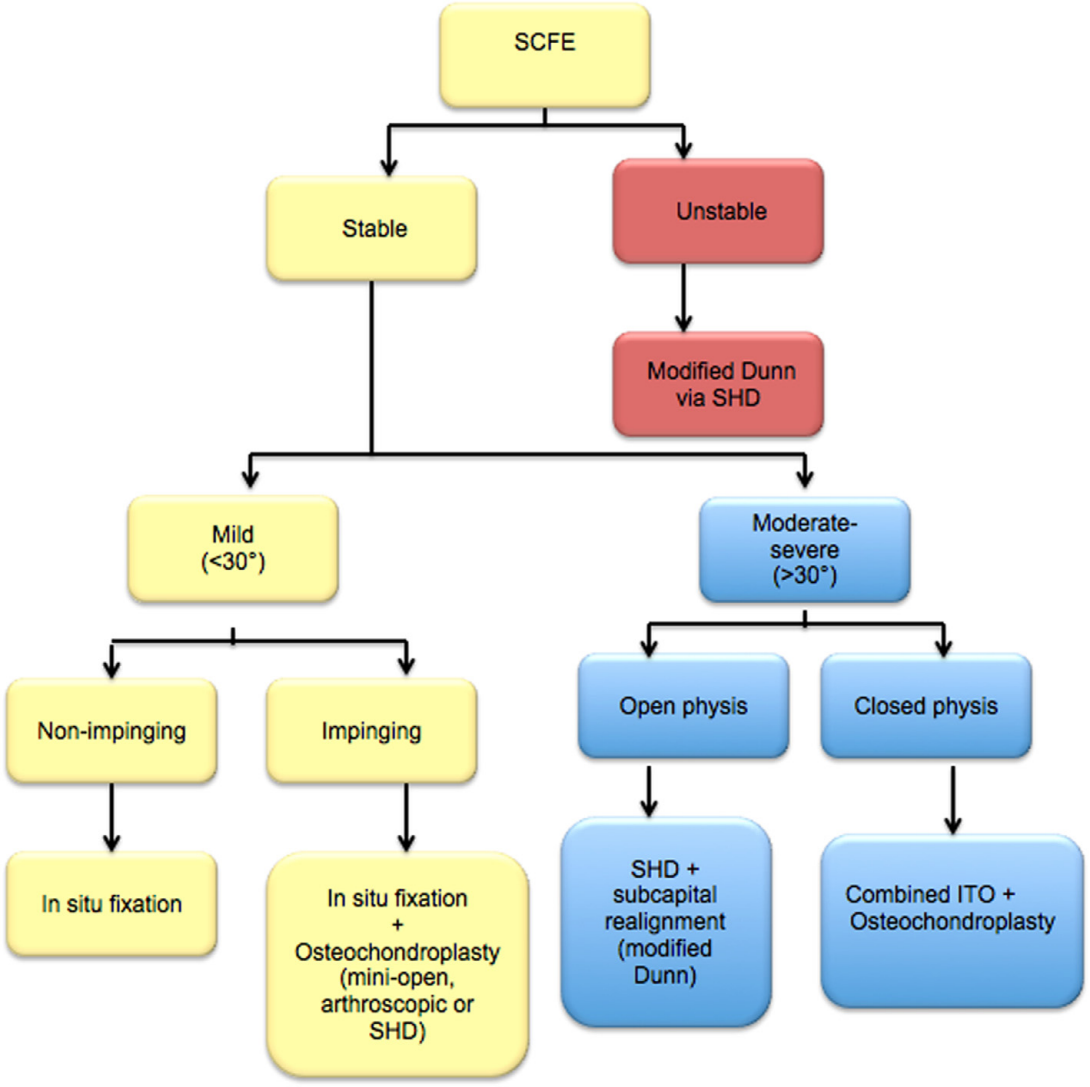
The authors’ proposed algorithm for management of slipped capital femoral epiphysis.

Based on our institutional practice and the literature review, we developed an algorithm for management for patients with SCFE (Figure 1). This algorithm would actively address impingement in hips with SCFE in order to prevent or delay OA, while considering substantial safety regarding the risk of ON. We adopt a skillful neglect strategy for clinically asymptomatic hips with radiographic signs of impingement.

During the period from 2013 to 2016, 125 surgical dislocations were performed, 75 were performed for SCFE. We consider surgical dislocation to be a safe, but precision procedure. In our earlier experience, patients with moderate to severe slips (slip angle more than 30°) were found to remain symptomatic with significant residual impingement after in situ pinning. Supported by the evidence of intraarticular damage from literature and from what we observed intraoperatively when we later performed SHD to those hips, we recommend initial management of impingement in hips with SCFE (mild impinging, and all moderate and severe slips).

Unstable slips are managed on urgent basis (within 24 h) while stable slips are electively scheduled. Stable slips with radiographic signs of instability are protected from weight bearing and managed as unstable slips. Reduction maneuvers are not attempted, even in unstable slips, and no traction tables are used. Unstable slips are definitely managed by the modified Dunn procedure via SHD approach. In most cases, resection of the posterior callus after a retinacular flap was found adequate to reduce the head onto the neck without undue tension on the posterior retinaculum. This avoids the unnecessary routine neck shortening which might result in hip instability. Neck trimming is performed if still the head reduces with tension on the retinaculum. We found open reduction of unstable slips to be the best indication for SHD in patients with SCFE.

For patients with stable slips, the decision depends on both the slip angle and the presence of FAI. The lateral slip angle is measured according to the Southwick method (mild slips < 30° and moderate to severe > 30°). The lateral slip angle was found to correlate with clinical impingement better than the anterior slip angle. We define impinging slips on clinical basis as hip flexion of 90° or less, and internal rotation of 15° or less. For mild non-impinging slips, we perform in situ pinning using a single screw. For mild impinging slips, in situ pinning is performed along with OCP via mini-open anterior arthrotomy. We consider arthroscopic osteochondroplasty a valid alternative.

For moderate to severe stable slips, the decision will depend on the state of physeal maturity. Slips with open physis are managed by the modified Dunn procedure via SHD approach. The presence of an open physis makes mobilization of the epiphysis easier with less potential harm to the retinacular vessels. In slips with partially closed or closed physis (computed tomography (CT) scan sometimes mandatory), we found subcapital realignment (modified Dunn) to be a high-risk procedure with significant threat to epiphyseal vascularity. Thus, we attempt the more distal ITO with flexion, valgus and derotation components tailored according to the degree of ROM loss. We perform the ITO through SHD. The SHD approach was found to be beneficial in many aspects. First, it provides good visualization to the proximal femur, metaphyseal bump, and the lesser trochanter, allowing protection of the main trunk medial femoral circumflex artery (MFCA) during an ITO. Second, it allows dynamic impingement test, which we routinely perform after the osteotomy to assess impingement, and more precisely plan the ITO to decide the adequacy of OCP. Third, it allows inspection and treatment of intra-articular pathologies. Fourth, we distalize the trochanteric flip to restore the abductor muscle tension and compensate for the functional coxa vara resulting from continued trochanteric overgrowth.

## Conclusion

Understanding of the role of impingement in patients with SCFE is still evolving. Osteonecrosis is by far the worst complication in a patient with SCFE. The symptomatology of SCFE is related to the multiplanar nature of the deformity and resultant FAI. Whether symptomatic or asymptomatic, FAI needs to be addressed by either surgical or expectant strategy. Even mild slips can be clinically impinging and predisposing to OA. Physeal maturity influences the choice of osteotomy level with, subcapital realignment for open physes and intertrochanteric osteotomies for closed physes. Prospective multicenter studies are still necessary to determine the best approach in treatment and delay the onset and progression of osteoarthritis.

## Conflict of interest

No benefits in any form have been received or will be received from a commercial party related directly or indirectly to the subject of this article. All authors declare no conflict of interest.
